# In Vitro Investigation of Therapy-Induced Senescence and Senescence Escape in Breast Cancer Cells Using Novel Flow Cytometry-Based Methods

**DOI:** 10.3390/cells13100841

**Published:** 2024-05-15

**Authors:** Fanni Tóth, Zahra Moftakhar, Federica Sotgia, Michael P. Lisanti

**Affiliations:** 1 Translational Medicine, University of Salford, Salford M5 4WT, UK; ftoth@cemm.at (F.T.);; 2 The CeMM Research Center for Molecular Medicine of the Austrian Academy of Sciences, Lazarettgasse 14, AKH BT 25.3, 1090 Wien, Vienna, Austria

**Keywords:** breast cancer, therapy-induced senescence, senescence escape, flow cytometry

## Abstract

Although cellular senescence was originally defined as an irreversible form of cell cycle arrest, in therapy-induced senescence models, the emergence of proliferative senescence-escaped cancer cells has been reported by several groups, challenging the definition of senescence. Indeed, senescence-escaped cancer cells may contribute to resistance to cancer treatment. Here, to study senescence escape and isolate senescence-escaped cells, we developed novel flow cytometry-based methods using the proliferation marker Ki-67 and CellTrace CFSE live-staining. We investigated the role of a novel senescence marker (DPP4/CD26) and a senolytic drug (azithromycin) on the senescence-escaping ability of MCF-7 and MDA-MB-231 breast cancer cells. Our results show that the expression of DPP4/CD26 is significantly increased in both senescent MCF-7 and MDA-MB-231 cells. While not essential for senescence induction, DPP4/CD26 contributed to promoting senescence escape in MCF-7 cells but not in MDA-MB-231 cells. Our results also confirmed the potential senolytic effect of azithromycin in senescent cancer cells. Importantly, the combination of azithromycin and a DPP4 inhibitor (sitagliptin) demonstrated a synergistic effect in senescent MCF-7 cells and reduced the number of senescence-escaped cells. Although further research is needed, our results and novel methods could contribute to the investigation of the mechanisms of senescence escape and the identification of potential therapeutic targets. Indeed, DPP4/CD26 could be a promising marker and a novel target to potentially decrease senescence escape in cancer.

## 1. Introduction

Cellular senescence has been described as the irreversible arrest of cell proliferation in response to different types of cellular stress [1,2,3]. The activation of the senescence programme is a natural intrinsic response to cancer treatment in addition to the desired apoptotic response of tumour cells [1,3,4,5], and therapy-induced senescence (TIS) has been documented in various types of human malignancies [6,7]. Breast cancer is among the most studied types of cancer in TIS research. A wide range of chemotherapeutic drugs has been reported to induce senescence in vitro in breast cancer cells, such as doxorubicin, etoposides, irinotecan, methotrexate, paclitaxel, cisplatin, 5-fluorouracil and palbociclib [8,9,10,11,12,13]. Moreover, the induction of senescence has been detected in tumour tissues of breast cancer patients after receiving chemotherapy, indicating that senescence is a clinically relevant response to cancer therapy [14,15,16,17]. In early experiments, therapy-induced senescence (TIS) was considered to have a beneficial function in cancer treatment. However, according to recent studies on different types of cancers, the detrimental effects of TIS outweigh its beneficial effects and could ultimately contribute to therapy resistance and tumour recurrence [12,18,19,20,21,22]. In fact, the increased expression of senescence markers has been associated with tumour recurrence and poor overall survival of breast cancer patients [23], and the elimination of therapy-induced senescent cells decreased metastasis and tumour relapse in murine breast cancer models [24]. Currently, there are several ongoing clinical trials investigating the effect of senolitycs in combination with chemotherapy in triple-negative breast cancer patients [25].

Although cellular senescence has been defined as an irreversible form of cell cycle arrest, now it is known that the halted proliferation of cancer cells is not always terminal, and some senescent cells can restore their proliferation capacity, implying the dynamic nature of senescence in cancer [26,27]. The emergence of senescence-escaped cells in TIS models was reported by several research groups, challenging the definition of senescence [21,28,29,30,31,32,33,34,35,36]. Besides these reports, there are other studies demonstrating the emergence of senescent cells that were able to resume proliferation, termed senescent-like cells, premature, reversible, or incomplete senescence, leading to confusion regarding the terminology of senescence escape [37,38,39]. Models for senescence escape in cancer are mostly based on studies of heterogeneous cell cultures, where the origin of the senescence-escaped cell population cannot be precisely identified. To overcome this limitation of senescence escape studies, senescent cells have been isolated based on their increased cell size and senescence-associated β-galactosidase (SA-β-gal) expression through the use of fluorescence-activated cell sorting (FACS), and it has been demonstrated that this sorted cell population was able to escape from senescence and generate tumours in vivo [40,41,42]. Furthermore, Was et al. (2017) used a time lapse technique to monitor senescence escape and demonstrated that the proliferating cells emerging after senescence induction originated from senescent cells [43]. By analysing the gene expression of senescence-escaped cell colonies, it has also been confirmed that senescence-escaped cells did not originate from drug-resistant cells [31,32,34]. In general, the increasing number of cells and the colony formation ability of the cells have been used to detect and evaluate senescence escape, which requires a prolonged incubation time and results in a mixture of senescent and senescence-escaped cells [31,34,42,44].

Here, we investigated senescence escape in different in vitro breast cancer models and established a flow cytometry-based method to improve the detection and quantification of senescence escape. By using this method, we investigated the effect of a novel senescence marker (DPP4/CD26) and a senolytic drug (azithromycin) on the senescence-escaping ability of breast cancer cells. For further characterisation of senescence escape, we established novel FACS-based live cell sorting method and tested different functional assays using the isolated senescence-escaped cells.

## 2. Materials and Methods

### 2.1. Cell Lines and Cell Culturing

MCF-7, MDA-MB-231 and MCF-10A cells were obtained commercially from the ATCC. MCF-7 and MDA-MB-231 breast cancer cells were maintained in Dulbecco’s Modified Eagle Medium, High glucose (DMEM, Sigma, Poole, UK) supplemented with 10% Heat-Inactivated (HI) FBS (Gibco, Paisley, UK), 1% Glutamax (100X, Gibco, cat. number: 35050061, and 1% Penicillin–Streptomycin (Gibco). MCF-10A breast epithelial cells were maintained in Mammary Epithelial Cell Growth Medium (MEGM, Lonza, Walkersville, MD, USA, cat. number: CC-3150). All cell lines were maintained in a humidified incubator at 37 °C and 5% CO_2_.

### 2.2. Senescence Induction and Treatments

The cells were seeded at an empirically determined density that allowed logarithmic growth during the senescence-inducing period. For senescence induction in MCF-7 and MDA-MB-231 cells, the cells were treated with bromodeoxyuridine (BrdU), gemcitabine (GEM) or palbociclib (PALBO). The concentrations and incubation times were determined empirically for both cell lines by monitoring the alteration of cell morphology, growth arrest and cell death (apoptotic cell morphology, detachment of the cells), to induce a relatively homogeneous senescence phenotype without the induction of a substantial amount of cell death. Moreover, previous in vitro studies [38,45] and the clinical administration of the drugs were also considered for the duration of GEM and PALBO treatment [46,47,48]. Seeding density was optimised for both cell lines to maximise the number of senescence-induced cells but avoid the induction of a quiescence-like state due to contact inhibition. For senescence induction in MCF-7 cells: 48 h after seeding (1 × 10^5^ cells/well in 6 well plates), the cells were treated with 5 µM BrdU for 7 days or 500 nM PALBO for 14 days, applying the treatment with every medium change (every 2–3 days), or treated with 100 nM GEM for 24 h and incubated without GEM for 7 days. For senescence induction in MDA-MB-231 cells: 24 h after seeding (2 × 10^5^ cells/well in 6 well plates), the cells were treated with 50 µM BrdU for 7 days, 200 nM GEM for 7 days or 1 µM PALBO for 14 days, applying the treatment with every medium change. BrdU, PALBO and AZI were dissolved in DMSO; GEM, CQ and SITA were dissolved in MQ water; all compounds were purchased from Sigma-Aldrich.

### 2.3. SA-β-Galactosidase Staining

The expression of SA-β-galactosidase was detected using the Senescence β-galactosidase Staining Kit (Cell Signaling) following the manufacturer’s instructions. The cells were seeded in 96-well optical bottom plates (Thermo Fisher Scientific, Waltham, MA, USA, cat. number: 165305) at a density of 1 × 10^4^ cells/well. After 48 h, the medium was removed, and the cells were fixed with Fixative Solution (provided in the kit) and incubated with the working solution for 6 h at 37 °C without CO_2_. After incubation, the working solution was removed, and the cells were stained with 1 µM Hoechst solution (Thermo Fisher) and imaged using the EVOS FL Auto Imaging System (Thermo Fisher Scientific, cat. number: AMF7000). The images were analysed using ImageJ software. The percentage of SA-β-galactosidase-expressing cells was calculated as the number of SA-β-gal-positive cells divided by the total cell number based on X-gal and Hoechst staining (Figure S1).

### 2.4. ELISA (Enzyme-Linked Immunosorbent Assay)

To collect the culture medium samples, the cells were incubated in a drug-free medium for 4 days. The culture medium was centrifuged (1000× *g*, 10 min, 6 °C), and the supernatant was collected and used immediately or stored at −80 °C. The levels of IL-6 and IL-8 were detected using ELISA kits (Thermo Fisher Invitrogen, IL-6 cat. number: KHC0061, IL-8 cat. number: KHC0081) according to the manufacturer’s instructions. Absorbance was read using a plate reader (Thermo Fisher, Varioskan™ LUX microplate reader) at 450 nm, and background signals were measured from the fresh culture medium and subtracted from each value. The results were quantified by using a standard logarithmic curve and normalised by the cell numbers.

### 2.5. Immunofluorescence Staining

The cells were seeded in 96-well optical bottom plates (Thermo Scientific) at a density of 1 × 10^4^ cells/well, and after 48 h, the cells were fixed in 4% paraformaldehyde (Thermo Fisher, PFA diluted in PBS), permeabilised with 0.3% (*v*/*v*%) Triton-X100 (Sigma) diluted in 1% (*w*/*v*%) BSA (Sigma) and incubated with 1% BSA for 30 min at room temperature. For BrdU staining, DNA was denatured via 20 min incubation with 1 M HCl at 40 °C, and the samples were washed twice with 0.1 M borate buffer pH 8.0 for 10 min to neutralise the pH. The cells were labelled with primary antibodies against Ki67 (1:250, SP6, 300 µg/mL, Thermo Fisher Invitrogen, cat. number: MA5-14520), BrdU (1:50, IIB5, 200 µg/mL, Santa Cruz Biotechnology, Santa Cruz, CA, USA, cat. number: sc-32323) or γH2AX phospho-S139 (1:500, 9F3, 1 mg/mL, Abcam, Cambridge, UK, cat. number: ab26350) diluted in 1% BSA for 1 h at room temperature, and incubated with secondary antibodies (1:1000, Alexa Fluor Plus 488—anti-mouse, Alexa Fluor 594—anti-rabbit, Alexa Fluor 594—anti-mouse, 2 mg/mL, Thermo Fisher Invitrogen) diluted in 1% BSA for 30 min at room temperature. Afterwards, the cells were incubated with 1 µM Hoechst solution (Thermo Fisher) and imaged using the EVOS FL Auto Imaging System. To image the cells with higher magnification (40X), the cells were seeded on round coverslips (15 mm diameter) in a 24-well plate at a density of 4 × 10^4^ cells/wells. After staining, the coverslips were mounted on glass slides using 10 µL mounting medium with DAPI staining (Vector labs). The images were analysed with ImageJ software (v1.53).

### 2.6. Immunostaining for Flow Cytometry

The cells were collected, fixed in ice-cold 70% (*v*/*v*%) ethanol, and incubated at −20 °C overnight. The samples were thawed, washed with 1% BSA (*w*/*v*%, dissolved in PBS), and incubated with primary antibodies (100 µL/2–5 × 10^5^ cells) against γH2AX (1:500, phospho S139, 9F3, 1 mg/mL, Abcam) or Ki-67 (1:250, SP6, 300 µg/mL, Thermo Fisher Invitrogen) diluted in 1% BSA for 30 min at room temperature. The cells were incubated with secondary antibodies (1:1000, Alexa Fluor Plus 488—anti-mouse; Alexa Fluor 660—anti-rabbit, 2 mg/mL, Thermo Fisher Invitrogen) diluted in 1% BSA for 30 min at room temperature and resuspended in 1% BSA with a concentration of 5 × 10^5^ cells/500 µL. For DPP4/CD26 staining, the cells were collected and resuspended in 1% BSA dissolved in PBS with a concentration of 3–5 × 10^5^ cells/sample and stained with 100 µL staining solution with CD26-PE antibody (1:100, 2A6, 0.1 mg/mL, Invitrogen) diluted in 1% BSA for 30 min on ice. The cells were washed and resuspended in 1% BSA. 2 × 10^4^ single cells were recorded using the Attune NxT Flow Cytometer. The results were analysed using FlowJo software (v10.8.0).

### 2.7. Cell Cycle Analysis

Cell cycle analysis was performed via flow cytometry using the double staining of Ki-67 and propidium iodide (PI) nuclear DNA stain. After immunostaining with Ki-67 antibody (described above), the cells were stained with Muse™ Cell Cycle Assay buffer (200 µL/5 × 10^5^ cells) containing PI stain (Luminex, Austin, TX, USA) for 20 min at room temperature, protected from light. Then, 300 µL/5 × 10^5^ cells of PBS was added to each sample, and 2 × 10^4^ single cells were recorded using the Attune NxT Flow Cytometer while applying compensation. The samples were manually categorised into cell cycle stages and analysed using FlowJo software.

### 2.8. SRB (Sulphorhodamine B) Assay

For this, 1 × 10^4^ cells/100 µL were seeded in 96-well plates. For growth assays, the cells were incubated without changing the medium and fixed after 24, 48, 72 and 96 h. For drug treatment assays, 24 h after seeding, the medium was replaced, and the cells were treated with selected compounds using the concentrations and incubation times indicated in each experiment. The cells were washed with PBS, fixed with 10% (*v*/*v*%) trichloroacetic acid (TCA) for 1 h at 4 °C and stained with 0.4%(*w*/*v*%) Sulphorodamine B (SRB, Sigma) dissolved in 1% (*v*/*v*%) acetic acid for 15 min. The cells were washed three times with 1% acetic acid and dried for at least 2 h. The incorporated dye was dissolved in 10 mM Tris-HCl pH 8.8 buffer (Trizma Base, Sigma) solution, and the absorbance was read using a plate reader (Thermo Fisher, Varioskan™ LUX microplate reader) at 565 nm.

### 2.9. Silencing of DPP4/CD26 Expression

DPP4 expression was silenced by using the Lenti-PacTM HIV Expression Packaging Kit (Genecopoeia) following the manufacturer’s instructions. Briefly, 1.5 × 10^6^ 293Ta packaging cells (human embryonic kidney cells) were seeded in T75 flasks and transfected with lentiviral vectors encoding three different clones for DPP4 siRNA (HSH004434-LVRV6GP-a, HSH004434-LVRV6GP-b, HSH004434-LVRV6GP-c) and a scrambled vector (HSH004434-LVRV6GP), using 2.5 µg lentiviral plasmid, 5.0 µL Lenti-Pac HIV mix and 15 µL of EndoFectin Lenti reagent diluted in Opti-MEM (Gibco). The cells were incubated overnight, and the medium was replaced with 10 mL fresh medium supplemented with 20 µL of TiterBoost reagent. Lentivirus-containing culture medium was collected, centrifuged (10 min, 500× *g*), filtered through a 0.45 µm filter and stored at −80 °C or used immediately. For transduction, 5 × 10^5^ MCF-7 or MDA-MB-231 cells were seeded in T25 flasks and cultured until they reached 70–80% confluence. Then, 3 mL of lentivirus-containing medium diluted with 3 mL of culture medium and 5 µg/mL polybrene (Santa Cruz) was added to the cells, and the cells were incubated overnight. Afterwards, the cells were cultured in fresh medium until they reached 80–90% confluence. The successfully transduced cells were selected with 1 µg/mL (empirically determined concentration) puromycin (Sigma) until the mock control (not transduced) cells were eliminated. Cells transduced with the HSH004434-LVRV6GP-a clone were selected to be used in subsequent experiments, labelled DPP4 siRNA. Cells transduced with scrambled vector were used as experimental control, labelled ctrl siRNA.

### 2.10. CellTrace CFSE (Carboxyfluorescein Succinimidyl Ester) Staining and FACS

After senescence induction, the cells were washed with DPBS+Ca^2+^/Mg^2+^(Gibco) and incubated with 5 µM CellTrace CFSE dye (Thermo Fisher) diluted in DPBS+Ca^2+^/Mg^2+^ for 20 min in the incubator. Afterwards, the staining solution was removed, and the cells were washed with culture medium and cultured in fresh medium for 10 (senescent cells) or 4 (control cells) days in the incubator. After CellTrace CFSE staining, the cells were collected, centrifuged, resuspended in pre-sort buffer (BD Biosciences, Franklin Lakes, NJ, USA, cat. number: 563503) at a concentration of 1 × 10^6^ cells/mL and sorted using the Sony SH800S Cell Sorter. The gates were adjusted to separate senescent and senescence-escaped cells by using control and senescent cells stained with CellTrace CFSE.

### 2.11. Migration Assay

After sorting, the cells were seeded in Falcon™ Cell Culture Inserts (PET membrane with 8 µm pores, for 24 well plates) at a concentration of 5 × 10^4^ cells (MCF-7) or 1 × 10^4^ cells (MDA-MB-231) in 500 µL medium without FBS and placed in the wells of a 24-well plate filled with 500 µL of medium with (MDA-MB-231) or without (MCF-7) 10% FBS. MCF-7 cells were serum-starved for 4 h, and then the medium in the bottom of the 24-well plates was replaced with medium with 10% FBS. The inserts were incubated for 24 h (MCF-7) or overnight (MDA-MB-231) in the incubator. Medium and cells were removed from the internal side of the inserts using a cotton swab, and the inserts were placed in 70% (*v*/*v*%) ethanol for 10 min to fix the migrated cells on the external side of the insert. The cells were stained with 0.2% (*v*/*v*%) crystal violet solution (Sigma) diluted in 70% ethanol for 30 min at room temperature. The staining solution was removed, and the inserts were washed with water and dried overnight. The membranes were imaged using the EVOS FL Auto Imaging System (Thermo Fisher), and images were analysed with ImageJ software.

### 2.12. Mammosphere Formation Assay

In this step, 6-well plates were coated with 2 mL/well of poly-HEMA solution (12 g of 2-hydroxyethylmethacrylate (Sigma) dissolved in 1 L of 95% ethanol), and incubated for 3 days at 50 °C. After sorting, the cells were seeded in the coated plates at a density of 5000 cells/well in mammosphere medium (DMEM-F12 medium without phenol red (Gibco) complemented with 2% (*v*/*v*%) B-27 supplement (Gibco), 20 ng/mL EGF (Invitrogen) and 1% Penicillin–Streptomycin (Gibco)) and incubated for 5 days in the incubator. Mammospheres bigger than 50 µm were manually counted using an eye piece graticule of the microscope.

### 2.13. Colony Formation Assay

For the colony formation assay, the cells were seeded in 6-well plates filled with 2 mL of culture medium with a density of 1000 cells/well (MCF-7) or 500 cells/well (MDA-MB-231) and incubated in the incubator for 14 days. Afterwards, the cells were fixed in 70% ethanol for 10 min at room temperature and stained with 0.5% (*v*/*v*%) crystal violet solution (Sigma) diluted in 70% ethanol for 30 min at room temperature. The staining solution was removed, and the plates were washed with water and dried overnight. The plates were imaged using the EVOS FL Auto Imaging System (Thermo Fisher), and the images were analysed with ImageJ software.

### 2.14. Statistical Analysis

All of the experiments were repeated at least 3 times with 2–3 technical replicates, or as indicated in figure legends. Data normality was assessed using the Shapiro–Wilks test. For statistical analysis of pairwise comparisons (two groups), a two-tailed unpaired *t*-test was used with statistical significance set at *p* < 0.05. For multiple group comparisons (3 < groups), a one-way ANOVA test corrected with Dunnett’s multiple comparison test was used, with statistical significance set at *p* < 0.05. All of the statistical analyses were performed using GraphPad Prism software (version 7).

## 3. Results

### 3.1. Senescence Induction and Characterisation of Senescent MCF-7 and MDA-MB-231 Cells

**Figure 1:**
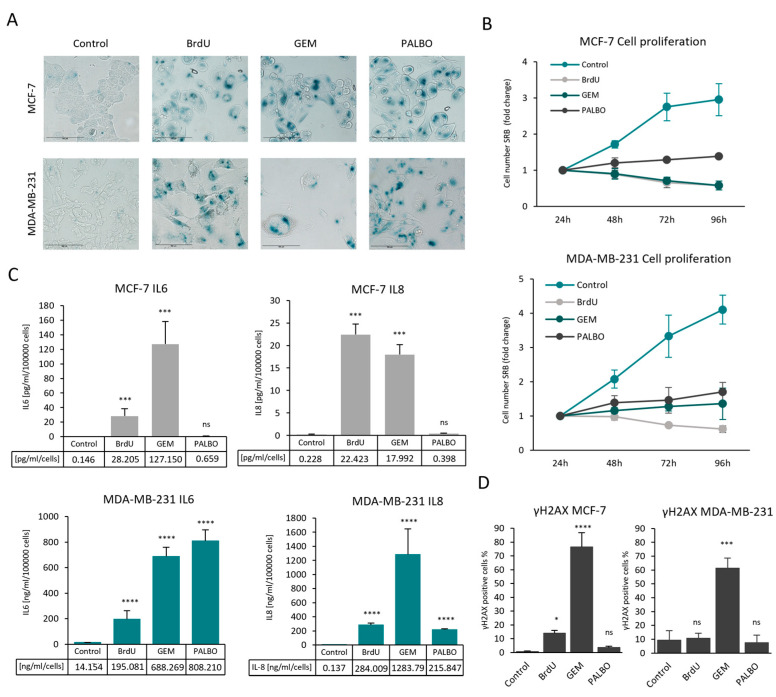
Senescence induction in MCF-7 and MDA-MB-231 cells using bromodeoxyuridine (BrdU), gemcitabine (GEM) and palbociclib (PALBO). (**A**) Representative images of control and senescent MCF-7 and MDA-MB-231 cells. Cells were fixed and stained with X-gal and imaged using EVOS at 20× magnification; scale bars indicate 100 µm. The blue colour indicates SA-β-galactosidase activity. (**B**) Cell proliferation of control and senescent MCF-7 and MDA-MB-231 cells was measured using SRB assay at different time points; the values were normalised to the baseline values measured 24 h after cell seeding and represented as fold change. Graph represents the mean values of two independent experiments ± SEM. (**C**) The secretion of IL-6 and IL-8 was measured using the ELISA assay. The cells were seeded in 6-well plates and cultured for 4 days before collecting the culture medium containing the secreted cytokines. The graphs represent the amount of IL-6 and IL-8 quantified by known concentrations of standards and normalised by cell numbers. (**D**) The expression of γH2AX is represented as the percentage of positively stained cells compared to the total population. The gates for γH2AX-positive cells were adjusted by using unstained cells. Bar graphs represent the mean of three independent experiments ± SEM. Statistical significance (in relation to control): ns *p* > 0.05; * *p* ≤ 0.05; *** *p* ≤ 0.001; **** *p* ≤ 0.0001.

To establish an in vitro cellular model, we used two breast cancer cell lines (MCF-7, MDA-MB-231), which have been widely used for investigating drug-induced senescence and senescence escape [28,33,49,50,51,52]. MCF-7 and MDA-MB-231 cells were treated with bromodeoxyuridine (BrdU), gemcitabine (GEM) and palbociclib (PALBO), which have been described to induce senescence via different pathways [53,54,55]. The optimal drug concentrations and incubation times for senescence induction were empirically determined (Figure S2). Senescence induction was confirmed by the increased activity of lysosomal β-galactosidase, the induction of growth arrest and SASP (IL-6 and IL-8) secretion (Figure 1A–C). In MCF-7 cells, the secretion of IL-6 and IL-8 was significantly increased in BrdU- and GEM-induced senescent cells but not in PALBO-induced senescent cells, indicating a distinct senescence phenotype of PALBO-treated MCF-7 cells. MDA-MB-231 cells had a substantially higher basal expression of IL-6 and IL-8, which was significantly increased in all senescent MDA-MB-231 cells (Figure 1C). To investigate whether these treatments induce DNA damage in our models, the expression of γH2AX was analysed through the use of flow cytometry. In both MCF-7 and MDA-MB-231 cells, the GEM treatment significantly increased γH2AX expression, while BrdU- and PALBO-induced senescent cells were mostly negative for γH2AX staining (Figure 1D). 

**Figure 2:**
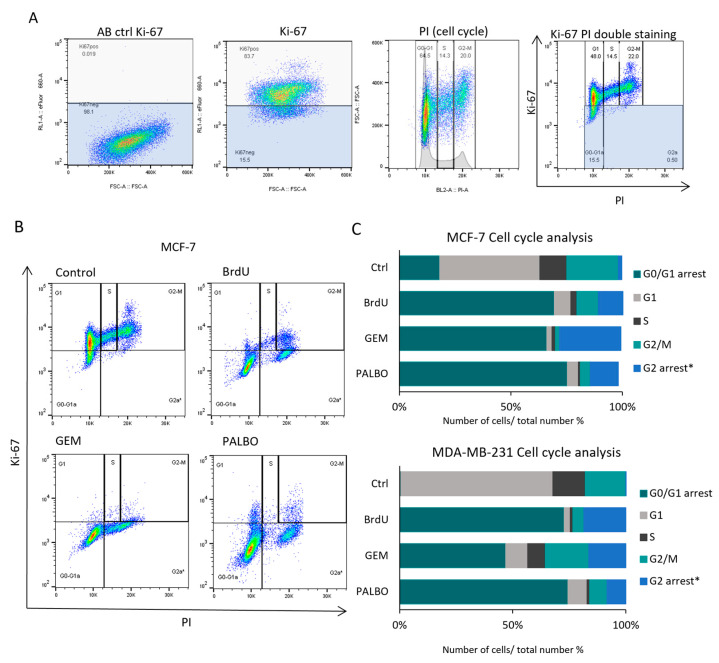
Cell cycle analysis of BrdU- GEM- and PALBO-induced senescent MCF7 and MDA-MB-231 cells using Ki-67 and PI staining. (**A**) Representative figures of control MCF-7 cells showing the gating strategy used to analyse cell cycle. Cells were manually categorised to cell cycle stages based on their DNA content detected using PI staining, and the gates for Ki-67-positive cells were adjusted by using AB ctrl cells (stained only with secondary antibodies). (**B**) Representative figure of the cell cycle analysis of control, BrdU-, GEM- and PALBO-induced senescent MCF-7 cells using Ki-67 and PI staining. (**C**) The bottom graph represents the distribution of cell cycle categories in control, BrdU-, GEM- and PALBO-induced senescent MCF-7 and MDA-MB-231 cells based on the mean values of three independent experiments. * The category of G2-arrested cells was based on the research of Miller et al. (2018) [58].

To further characterise the proliferation arrest in the senescent cells, the cells were co-stained with Ki-67 and propidium iodide (PI) (Figure 2). Ki-67 is known to be expressed in the nucleus during all active phases of the cell cycle but is absent from quiescent and senescent cells, while PI staining can differentiate between cell cycle stages [56,57]. Therefore, the Ki-67 and PI double staining method provides a more detailed cell cycle analysis by generating the following categories: G0/G1 arrest, G1 phase, S phase, G2/M phase and G2 arrest (Figure 2A). Based on our results, the number of cell-cycle-arrested (G1 or G2 arrest) cells was increased after senescence induction in MCF-7 and MDA-MB-231 cells, while the number of cells associated with active proliferation (G1-S-G2-M) was decreased (Figure 2B,C). In addition, GEM treatment also induced a significant increase in sub-G1 (in both MCF-7 and MDA-MB-231) and polyploid (in MDA-MB-231) populations (Figure S3).

### 3.2. The Senescence-Escaping Ability of the Cells Can Be Quantified by Ki-67 Staining, and It Is Dependent on the Cell Type and Mechanism of Senescence Induction 

**Figure 3:**
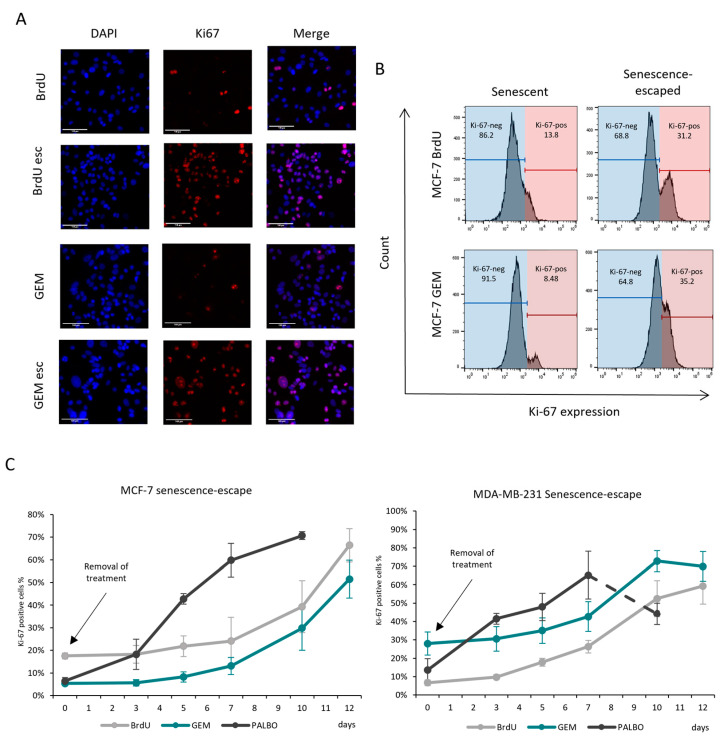
Actively proliferating senescence-escaped cells can be detected using Ki-67 staining. (**A**) Representative images demonstrating the increase in Ki-67 expression in BrdU- and GEM-induced senescence-escaped MCF-7 cells compared to the senescent cells. The cells were stained via immunostaining and imaged using EVOS at 20× magnification; scale bars indicate 100 µm. (**B**) Representative figures evaluating senescence escape in BrdU- and GEM-induced senescent MCF-7 cells based on Ki-67 expression measured using flow cytometry. The cells were categorised as Ki-67-negative (blue) and Ki-67-positive (red) populations. (**C**) Graphs show the evaluation of senescence escape in BrdU-, GEM- and PALBO-induced senescent MCF-7 and MDA-MB-231 cells based on Ki-67 expression measured using flow cytometry at different time points. Arrows indicate the removal of treatment, representing day 0. The graphs represent the mean values of three independent experiments ± SEM.

Because Ki-67 expression is highly decreased in growth-arrested/senescent cells, it could be used to detect cells that re-entered the cell cycle after senescence induction and thus escaped from senescence. To allow the cells to escape from senescence, senescent MCF-7 and MDA-MB-231 cells were incubated without a senescence-inducing drug for 10 days. Compared to senescent cells, senescence-escaped cells had an increased expression of Ki-67, which can be detected when using both immunostaining and flow cytometry (Figure 3A,B). By using the number of Ki-67-positive cells compared to the total cell number (expressed as the percentage of Ki-67-expressing cells), even small changes in Ki-67 expression can be accurately quantified using flow cytometry, allowing us to measure the senescence-escaping ability of cells (Figure 3B,C). Using this method, we compared the senescence-escaping ability of MCF-7 and MDA-MB-231 cells after inducing senescence via each drug. Based on the results, senescence-escaped cells started to appear around 5–7 days after the removal of BrdU/GEM. In contrast, PALBO-induced senescent cells escaped from senescence shortly after the removal of the PALBO treatment (Figure 3C and Figure S4). This indicates that the BrdU and GEM treatments induced a more stable but reversible cell cycle arrest compared to the PALBO treatment, which instead induced a more transient growth arrest. As a result, in subsequent experiments, senescence escape was assessed only in BrdU- and GEM-induced senescent cells.

### 3.3. Using Ki-67 Expression for the Quantification of Senescence Escape Revealed a Potential Role of Dipeptidyl-Peptidyl 4 (DPP4/CD26) in Senescence Escape 

**Figure 4:**
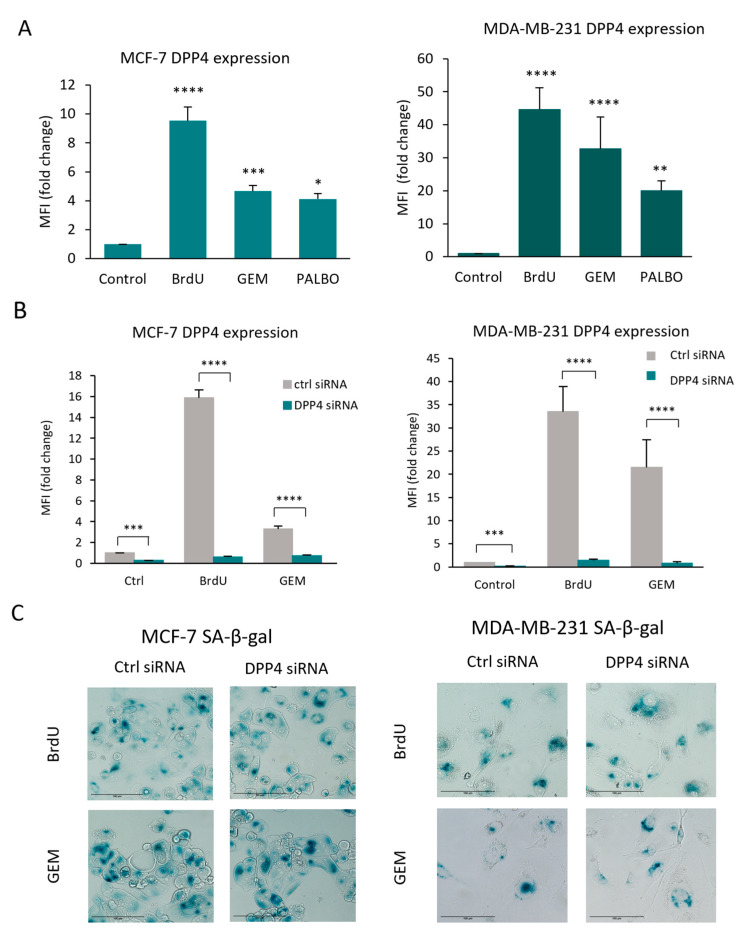
DPP4 expression is increased in BrdU-, GEM- and PALBO-induced senescent MCF-7 and MDA-MB-231 cells, but the silencing of DPP4 did not affect senescence induction. (**A**) The expression of DPP4/CD26 was measured using flow cytometry, represented as mean fluorescence intensity (MFI) of CD26-PE staining. The values were normalised to the MFI of non-senescent (control) MCF-7 and MDA-MB-231 cells. (**B**) The expression of DPP4 was silenced in both cell lines via lentiviral vector-mediated gene silencing, and the cells were labelled as DPP4 siRNA. Cells transduced with scrambled vector were used as experimental controls, labelled ctrl siRNA. DPP4 expression is represented as mean fluorescence intensity (MFI) of CD26-PE staining measured using flow cytometry, and the values were normalised to the MFI of non-senescent ctrl siRNA cells and represented as fold change. Bar graphs represent the mean values of three independent experiments ± SEM. Statistical significance: * *p* ≤ 0.05; ** *p* ≤ 0.01; *** *p* ≤ 0.001; **** *p* ≤ 0.0001. (**C**) Representative images of BrdU-, GEM- and PALBO-induced senescent ctrl siRNA and DPP4 siRNA cells. The cells were fixed and stained with X-gal and imaged using EVOS with 20× magnification; scale bars indicate 100 µm. The blue colour indicates SA-β-galactosidase activity.

DPP4/CD26 was identified as a marker of senescence in fibroblasts [59], and it has been described as a tumour promoter and tumour suppressor in various types of cancers [60]. However, its expression and potential function have not been previously investigated in senescent cancer cells. We found that in senescent MC-7 and MDA-MB-231 cells, the expression of DPP4/CD26 was significantly increased compared to the non-senescent cells, and the highest increase was detected after the BrdU treatment (Figure 4A). This BrdU-induced increase in DPP4/CD26 expression could also be detected in other breast cancer cell lines, which indicates that it could be a potential marker of senescence in breast cancer (Figure S5). To investigate the function of DPP4/CD26 in senescence, DPP4 expression was silenced in both cell lines, and senescence was subsequently induced by BrdU or GEM treatment (Figure 4B). We found that senescence could be induced by BrdU or GEM in DPP4-silenced cells similarly to DPP4 wild-type cells, which indicates that DPP4/CD26 expression is not essential for senescence induction or for the development of a senescence phenotype in MCF-7 and MDA-MB-231 cells (Figure 4C). 

**Figure 5:**
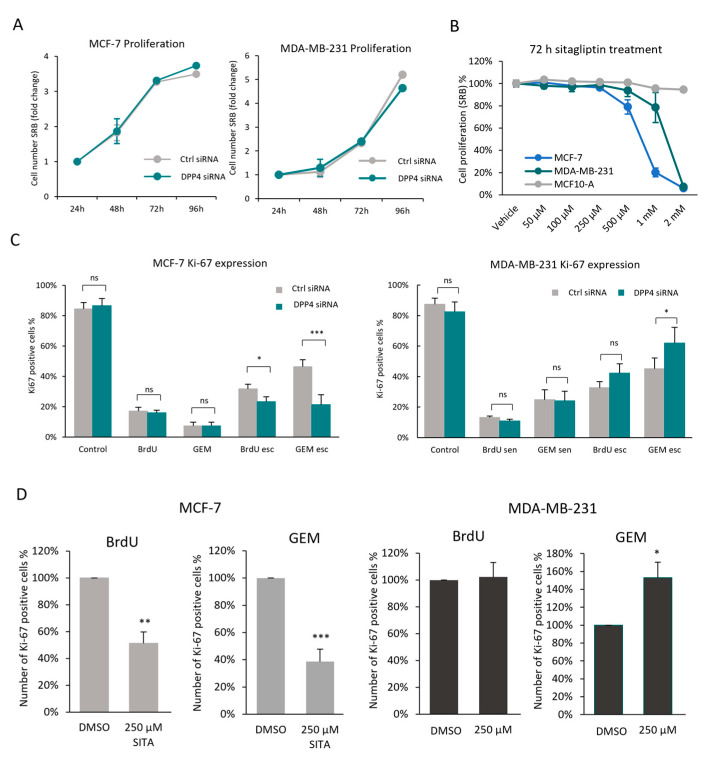
Effect of DPP4 silencing/inhibition on the senescence-escaping ability of MCF-7 and MDA-MB-231 cells. (**A**) Cell proliferation was measured using the SRB assay at different time points, and the values were normalised to the baseline values measured 24 h after cell seeding and represented as fold change. Graph represents the mean of three independent experiments ± SEM. (**B**) The cell viability of MCF-10A, MCF-7 and MDA-MB-231 cells was measured using the SRB assay after 72 h treatment of sitagliptin. Experiments were repeated three times with six technical replicates; error bars represent ± SEM. (**C**) Evaluation of senescence escape in ctrl siRNA and DPP4 siRNA cells based on Ki-67 expression measured via flow cytometry. (**D**) After senescence induction via BrdU and GEM treatments, MCF-7 and MDA-MB-231 cells were incubated for 10 days with sitagliptin treatment. The senescence-escaping abilities of the cells were assessed by the combination of Ki-67 expression and cell concentration represented as the number of Ki-67-positive cells. Values were normalised to DMSO-treated cells. Bar graphs represent the mean values of three independent experiments ± SEM. Statistical significance: ns *p* > 0.05; * *p* ≤ 0.05; ** *p* ≤ 0.01; *** *p* ≤ 0.001.

By using our previously established method for the quantification of senescence escape, we analysed the effect of DPP4 silencing or inhibition (by using sitagliptin treatment [61]) on senescence escape in MCF-7 and MDA-MB-231 cells (Figure 5). The chosen working concentration of sitagliptin (250 µM SITA) was based on its enzymatic activity reported in MCF-7 cells [62] and its toxicity in MCF-7, MDA-MB-231 and MCF10-A (normal epithelial control cells) (Figure 5B and Figure S6). Our results demonstrated that although DPP4 silencing or inhibition did not affect the cell proliferation or viability of senescent cells (Figure 5A,B), it showed a cell-type-dependent effect on the senescence-escaping ability of MCF-7 and MDA-MB-231 cells (Figure 5C,D). In both cell lines, Ki-67 expression was similarly decreased in DPP4 siRNA and ctrl siRNA cells upon senescence induction (Figure 5C). Interestingly, in BrdU- and GEM-induced senescent MCF-7 cells, DPP4 silencing significantly decreased senescence escape, whereas GEM-induced senescent MDA-MB-231 cells significantly increased senescence escape (Figure 5C). These observations were further confirmed by observing the inhibition of DPP4 activity (by SITA treatment), resulting in a similar cell-type-dependent decrease or increase in senescence escape (Figure 5D). Importantly, these results suggest a cell-type-dependent role of DPP4 activity in senescence escape. Moreover, these experiments demonstrate that the flow cytometry-based detection of senescence escape could be a useful method to identify proteins/pathways that play a role in the regulation of senescence escape.

### 3.4. DPP4 Inhibition Can Decrease Senescence Escape in MCF-7 Cells after Senolytic (Azithromycin) Treatment

**Figure 6:**
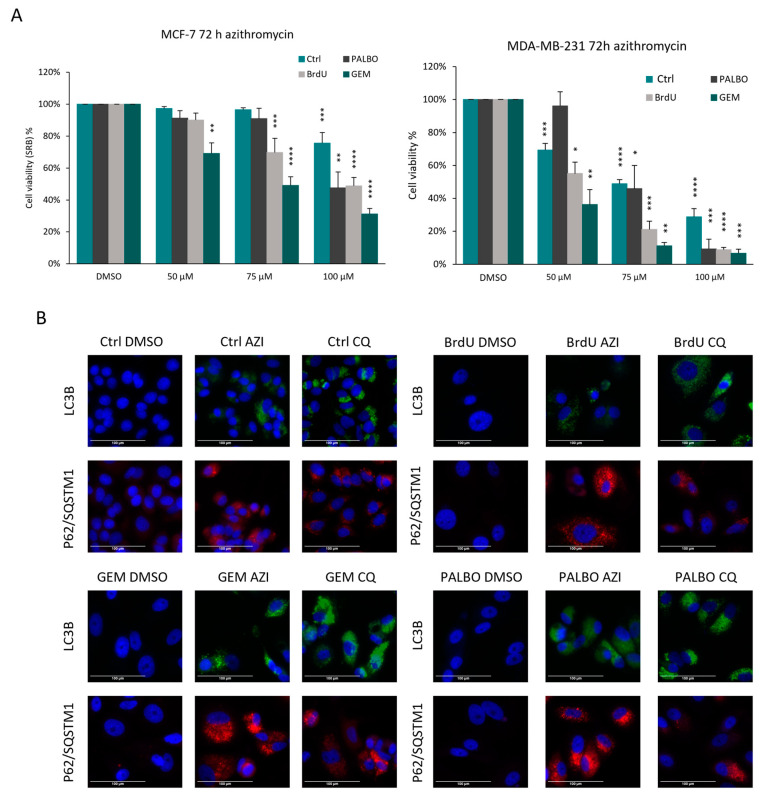
Azithromycin disrupts autophagy and has senolytic activity in senescent MCF-7 and MDA-MB-231 cells. (**A**) The cell viability of control, BrdU-, GEM- and PALBO-induced senescent MCF-7 and MDA-MB-231 cells was measured using the SRB assay after 72 h azithromycin treatment. Experiments were repeated three times with six technical replicates; error bars represent ± SEM. Statistical significance (in relation to control): * *p* ≤ 0.05; ** *p* ≤ 0.01; *** *p* ≤ 0.001; **** *p* ≤ 0.0001. (**B**) Representative images of LC3B and p62/SQSTM1 immunostaining of control, BrdU-, GEM- and PALBO-induced senescent MCF-7 cells after 48 h of 100 µM azithromycin and 50 µM chloroquine treatment. By using the same imaging settings for each condition, in untreated MCF-7 cells (DMSO), the signal intensity was below the detection threshold. The cells were imaged using EVOS at 20× magnification; scale bars indicate 100 µm.

Azithromycin has been previously identified by our group as a novel senolytic drug that selectively targets senescent fibroblasts [63]. However, its senolytic effect has not been tested in cancer cells. To test its potential senolytic effect, senescent MCF-7 and MDA-MB-231 cells were treated with azithromycin for 72 h using the same concentrations that had been used to target senescent fibroblasts (Figure 6). We found that azithromycin (AZI) treatment decreased the viability of senescent cells in both MCF-7 and MDA-MB-231 senescence models; however, PALBO-induced senescent cells were less sensitive to lower concentrations of AZI. The results also showed that MCF-7 cells are more resistant to AZI treatment compared to MDA-MB-231 cells, where the number of both senescent and non-senescent cells decreased (Figure 6A). Besides its well-known antibiotic effect, azithromycin has been described as having an impact on autophagic processes as well, which could be a potential mechanism explaining its senolytic effect [63,64]. To test this, we measured the expression of LC3B and p62/SQSTM1 in senescent MCF-7 and MDA-MB-231 cells after AZI treatment and compared it to a well-known autophagy inhibitor drug, chloroquine (CQ). Based on our results, both AZI and CQ resulted in an increase in LC3B and p62/SQSTM1 expression, indicating a similar effect on autophagic processes in both cell lines (Figure 6B). 

**Figure 7:**
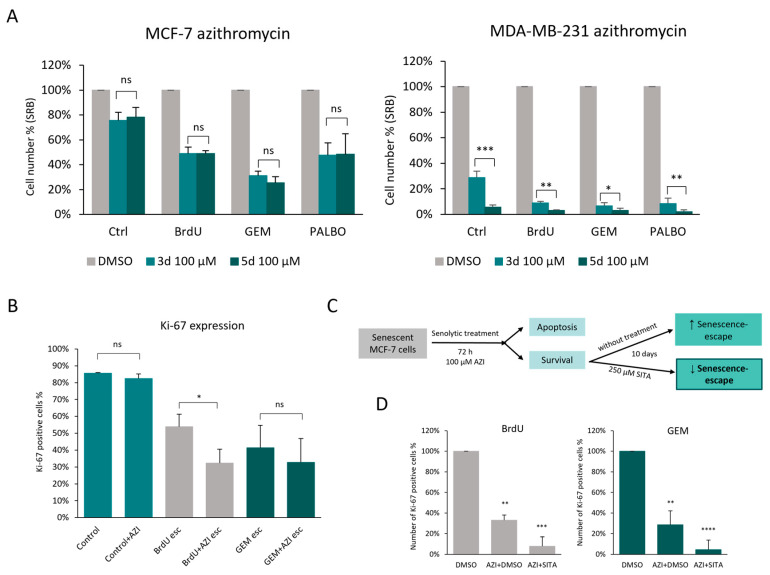
Senescence escape in azithromycin-treated senescent MCF-7 cells can be decreased via DPP4 inhibition (sitagliptin treatment). (**A**) The cell numbers of control, BrdU-, GEM- and PALBO-induced senescent MCF-7 and MDA-MB-231 cells were measured using the SRB assay after 3 days and 5 days of treatment with 100 µM azithromycin. Experiments were repeated three times with six technical replicates. (**B**) Control and senescent MCF-7 cells were treated with DMSO or 100 µM AZI for 72 h. Afterwards, the control cells were incubated 3 days without AZI, and senescent cells were incubated 10 days without AZI. The senescence-escaping ability of the cells was assessed via Ki-67 staining. Due to the reduced number of senescent cells after AZI treatment, the relative expression of Ki-67 has been used, referred to as Ki-67-positive cells (%). (**C**) Schematic figure representing the workflow of combination treatment using azithromycin and sitagliptin (**D**) The senescence-escaping abilities of the cells were assessed in terms of Ki-67 expression and cell concentration represented as the number of Ki-67-positive cells. Bar graphs represent the mean of three independent experiments ± SEM. Statistical significance (in relation to control): ns *p* > 0.05; * *p* ≤ 0.05; ** *p* ≤ 0.01; *** *p* ≤ 0.001; **** *p* ≤ 0.0001.

As demonstrated before, around 50% of senescent MCF-7 cells were resistant to the 72 h AZI treatment; therefore, the effect of 5 days of treatment was tested as well. We found that 5 days of AZI treatment did not further decrease the number of senescent MCF-7 cells, whereas it almost completely eliminated senescent and non-senescent MDA-MB-231 cells (Figure 7A). To test whether the surviving senescent MCF-7 cells are able to escape from senescence, the senescence-escaping abilities of AZI-treated cells were measured via Ki-67 using the previously described flow cytometry-based method (see Figure 3). After 72 h of AZI treatment, the surviving senescent cells were able to regain their proliferative capacity; however, in BrdU-induced senescent MCF-7 cells, senescence escape was decreased after AZI treatment (Figure 7B). After this, we tested whether the subsequential treatment with sitagliptin (SITA) would decrease senescence escape after AZI treatment in senescent MCF-7 cells (Figure 7C). Our results demonstrated that SITA treatment significantly decreased senescence escape and thus improved the senolytic effect of AZI in MCF-7 cells (Figure 7D).

### 3.5. FACS-Based Isolation Method Using CFSE Staining Allows the Functional Characterisation of Senescence-Escaped Cells

**Figure 8:**
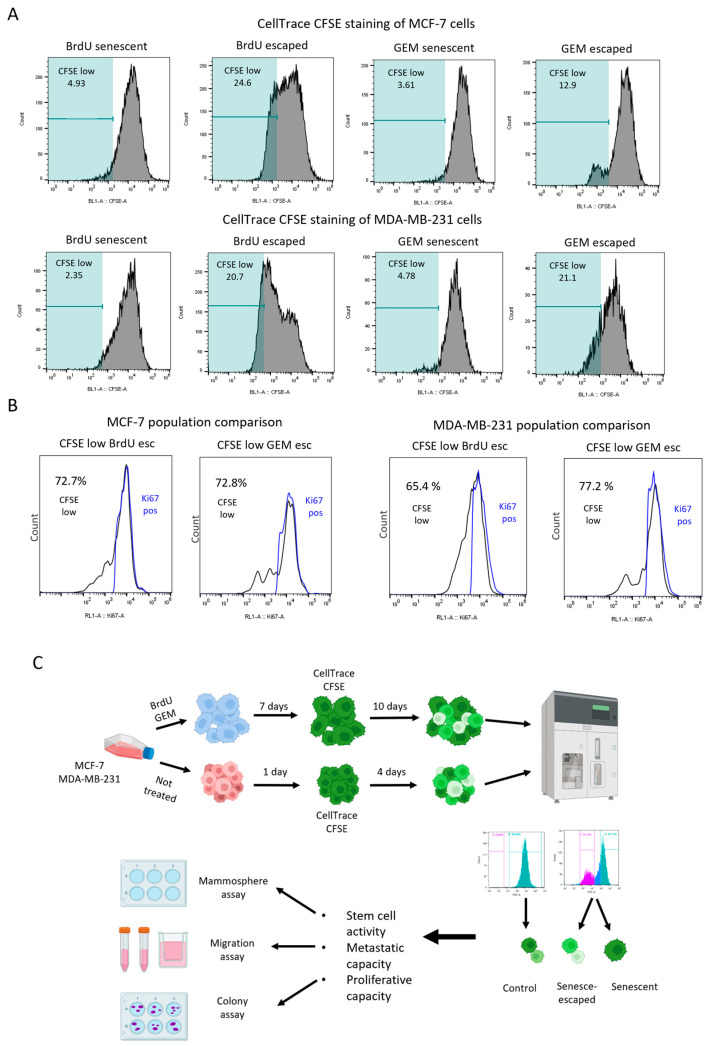
Senescence-escaped MCF-7 and MDA-MB-231 cells could be isolated using CFSE staining based on the loss of fluorescent signal due to cell proliferation. (**A**) Representative figures of the gating strategies for the isolation of senescence-escaped cells via flow cytometry using CFSE staining. The gates were adjusted manually to the basis of the histogram generated by the CFSE signal of BrdU- and GEM-induced senescent cells and cells with low expression of CFSE (CFSE low) were considered senescence-escaped cells. Figures were generated using FlowJo. (**B**) Representative figures of the results of population comparisons via FlowJo. The senescent cells were co-stained with CFSE and Ki-67, and the population of cells with low CFSE staining (CFSE low) were compared with the population of Ki-67-expressing cells (Ki-67 pos). The percentages represented next to the graphs indicate the correlation of the two populations. (**C**) The figure represents the workflow for the isolation of senescence-escaped cells by FACS. The figure was generated using BioRender.

Although analysing the expression of Ki-67 can be used to quantify senescence escape, the staining method requires the cells to be fixed, and thus, they can no longer be used for further functional characterisations. Therefore, we established a FACS-based method for the isolation/purification of senescence-escaped cells using a fluorescent dye known as CFSE (carboxyfluorescein succinimidyl ester), which covalently binds to intracellular proteins, and its signal intensity progressively decreases after each cell division [65]. By staining the cells with CFSE after senescence induction, the decreased intensity of CFSE staining was used to identify and isolate the actively proliferating senescence-escaped cells, while non-proliferative senescent cells retained high signal intensity (Figure 8A). To further validate the CFSE staining-based isolation of senescence-escaped cells, senescent cells were stained with CFSE, incubated for 10 days without the senescence-inducing drug (to allow senescence escape) and co-stained with Ki-67. The experiment demonstrated that most of the CFSE-low cells (senescence-escaped cells) were positive for Ki-67 staining, indicating that senescence-escaped cells were enriched in the CFSE-low population (Figure 8B). By using this FACS-based method, after 10 days of incubating senescent MCF-7 and MDA-MB-231 cells in a drug-free medium, both senescent and senescence-escaped cell populations were isolated for further functional assays, such as the mammosphere formation assay, migration assay and the colony formation assay (Figure 8C). Non-senescent cells were also isolated using CFSE staining and used as control cells.

**Figure 9:**
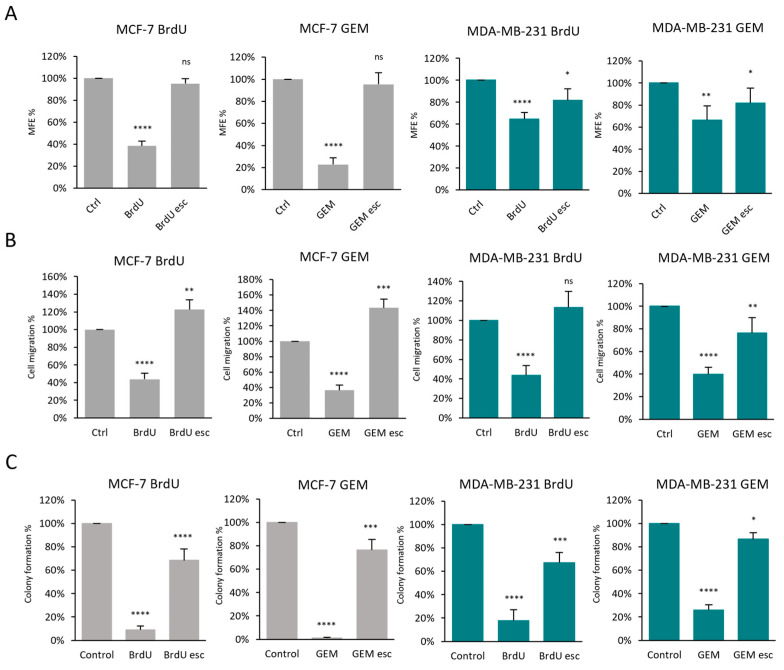
Investigating the effect of senescence escape in cancer progression using functional assays—stem cell activity, migration capacity and proliferative capacity. (**A**) The graphs represent the mammosphere formation efficiency (MFE) of control, senescent and senescence-escaped cells after cell sorting, normalised to the MFE of the control MCF-7 and MDA-MB-231 cells. (**B**) Cell migration was assessed using a transwell assay. Representative images of the migration are shown in Supplementary Figure S7A. The cells were stained with crystal violet and imaged using EVOS. To evaluate cell migration, the number of cells was counted with ImageJ using 4 images from each sample. (**C**) Proliferative capacity was assessed using the colony formation assay. Representative images of colony formation are shown in Supplementary Figure S7B. Cells were stained with crystal violet, and the number of colonies with more than 50 cells were counted using ImageJ. Experiments were repeated with three technical replicates; bar graphs represent the mean of three independent experiments ± SEM. Statistical significance (in relation to control): ns *p* > 0.05; * *p* ≤ 0.05; ** *p* ≤ 0.01; *** *p* ≤ 0.001; **** *p* ≤ 0.0001.

The results of these assays indicated that senescence-escaped cells regained the ability to proliferate, migrate and undergo anchorage-independent growth, which is associated with stem cell features (Figure 9 and Figure S7).

## 4. Discussion

A wide range of compounds have been described as being capable of inducing senescence in cancer cells through various molecular pathways, creating different phenotypes of senescent cells, which ultimately leads to a substantial variability of experimental results [39,66]. To carry out a thorough investigation of senescence in breast cancer, we used three different drugs (BrdU, GEM and PALBO), to potentially generate different senescent phenotypes in MCF-7 and MDA-MB-231 cells. BrdU is a synthetic analogue of the nucleoside thymidine, which has been described as inducing senescence in various cell types (including cancer cells) by inducing DNA damage or by being incorporated into specific AT-rich sequences and regulating the expression of senescence-associated genes [53,67,68]. According to our results, the BrdU treatment did not induce DNA damage in MCF-7 and MDA-MB-231 cells, indicating that it is more likely that it induced senescence via the upregulation of senescence-associated genes. Gemcitabine (GEM) is an analogue of deoxycytidine, and it incorporates into the newly synthetised DNA, causing DNA damage [69]. Although its activity has been extensively characterised, the senescence-inducing ability of GEM was only tested in pancreatic cancer cell lines [54,70,71]. In our in vitro model, GEM treatment induced DNA damage and senescence in both breast cancer cell lines. Palbociclib (PALBO) is a selective cyclin-dependent kinase 4/6 (CDK4/6) inhibitor that has been approved for advanced hormone receptor-positive and HER2-negative breast cancer treatment [46,72,73]. It has been proven in different cell types that PALBO treatment induced a reversible quiescence or a more stable senescence state, depending on which signalling pathways were activated after cell cycle arrest [38,74,75,76]. Based on the expression of senescence markers and the immediate regain of proliferative capacity, we suggest that the PALBO treatment induced a transient or reversible senescent phenotype in MCF-7 and MDA-MB-231 cells, which is different from the BrdU- or GEM-induced senescent phenotype. This phenomenon was observed in patient-derived glioma stem cells, human liver cancer cells and other breast cancer cells as well, and it was described as senescent-like quiescence or incomplete senescence [38,73,75]. Interestingly, in MCF-7 cells, the PALBO treatment induced a different senescent phenotype without the increased expression of IL-6 and IL-8. These differences in IL-6 and IL-8 expression in PALBO-induced senescent cells were reported by others as well, indicating that the increased expression of IL-6 and IL-8 induced by the PALBO treatment is cell-type-specific [75,77,78]. 

Although cellular senescence has been described as one of the main hallmarks of cancer [79], and there is growing evidence about its detrimental effect on the outcome of cancer therapies, the impact of therapy-induced senescence (TIS) is rarely considered in the clinical treatment of cancer. Despite significant progression in the in vitro identification and investigation of senescent cancer cells, clinically applicable diagnostic tests to detect senescent cells in cytological or histological samples with high levels of confidence are still lacking. Although several cell surface markers of cellular senescence have been identified for the detection of senescent cells, further validation of their specificity is required to exploit their diagnostic and therapeutic potential [59,80]. Dipeptidyl peptidase IV (DPP4/CD26) was identified as a cell surface marker for senescent fibroblast cells; however, it has not been validated in other cell types, such as in breast cancer cells [59]. DPP4/CD26 is a transmembrane glycoprotein with serine exopeptidase activity, and it regulates the activity of different bioactive peptides, including neuropeptides, growth factors, incretin hormones and cytokines as well [81]. Besides its enzymatic function, DPP4 can function as an adhesion molecule for proteins such as eADA (ecto-adenosine deaminase) and to extracellular matrix proteins such as collagen and fibronectin [82,83]. We found that the expression of DPP4/CD26 was significantly increased in BrdU-, GEM- and PALBO-induced senescent MCF-7 and MDA-MB-231 cells and in BrdU-treated breast cancer cell lines (CAMA-1, MDA-MB-468 and T47D), indicating that it could be a potential new marker of senescence in breast cancer. Moreover, cell surface markers could also be used as therapeutic targets for antibody-directed immunotherapies and for the administration of senolytic drugs by using antibody–drug conjugates [84]. For example, DPP4/CD26 and another cell surface marker of senescence, uPAR (urokinase plasminogen activator receptor), have already been described as potential therapeutic targets for the activation of antibody-dependent cell-mediated cytotoxicity and for CAR T cell (chimeric antigen receptor T cell) therapy against senescent cells in vitro and in vivo as well [59,85]. 

To establish a quantitative method for the evaluation of senescence escape, we measured the proportion of Ki-67-expressing cells after the drug-free incubation of senescent cells. To further investigate senescence-escaped cells with live-cell-based assays, we established a new method to isolate senescence-escaped cells through the use of FACS based on their restored proliferative capacity using CFSE staining. As already described, the limitation of studies about senescence escape is the lack of confirmation that the population of escaped cells originated from senescent cells, excluding the possibility that the examined cell population bypassed the senescent state. To resolve this issue, senescence models should be optimised by either generating a relatively homogeneous population of senescent cells (such as in our models) or by sorting the senescence-induced cells based on a senescence marker, such as increased granularity or β-galactosidase expression [40,41,42]. To achieve a better understanding of the cellular processes that regulate senescence escape, assessing the ability of cells to escape from senescence is essential. By using our established method, we revealed a potential cell-type-dependent role of DPP4/CD26 in the regulation of senescence escape by either decreasing (MCF-7) or increasing (MDA-MB-231) it. The inhibition of DPP4 (by sitagliptin treatment) resulted in a similar effect on the senescence escape of MCF-7 and MDA-MB-231 cells, indicating that senescence escape is regulated by the enzymatic activity of DPP4. However, further investigation should be conducted to determine the underlying mechanisms and molecular pathways that contribute to this effect of DPP4/CD24 activity.

Azithromycin has been described as a novel senolytic drug, selectively targeting senescent fibroblast cells; however, its effect has not been tested in senescent cancer cells [63]. Azithromycin (AZI) is a type of macrolide antibiotic that is used for the treatment of a wide range of bacterial infections [86]. In our experiments, AZI exhibited a senolytic effect in both cell lines; however, it was more effective in MDA-MB-231 cells. The assessment of autophagy-related protein expression revealed that AZI inhibited autophagy in senescent cancer cells, similar to chloroquine, which has already been used as a senolytic [87,88,89,90]. In fact, autophagy inhibition via AZI has also been reported by Renna et al. (2011), demonstrating that azithromycin treatment impaired autophagosome degradation via the inhibition of lysosomal acidification [64]. Thus, the increased sensitivity of MDA-MB-231 cells to azithromycin treatment can be explained by their increased dependence in terms of autophagy compared to MCF-7 cells, which have been categorised as autophagy-independent cells [91]. The population of senescent MCF-7 cells that were resistant to AZI treatment were able to escape senescence and continue to proliferate; however, the additional inhibition of DPP4 activity through the use of sitagliptin decreased senescence escape in the AZI-resistant senescent cells. The ability of senescent cells to escape after senolytic treatment has been described in different studies, where senescence-escaped cells that appeared after senolytic treatment exhibited increased cell proliferation and tumour formation in vitro and in vivo [31,43].

To further investigate senescence-escaped cells with live-cell-based assays, we established a method to isolate senescence-escaped cells through the use of FACS based on their restored proliferative capacity using CFSE live-cell staining. Although a similar staining method (using a membrane dye known as DiI) has been used to isolate senescence-escaped cells for functional assays [43], in most experiments, a prolonged incubation time (2–3 weeks) has been used to ensure that the number of senescence-escaped cells greatly outweighs the number of senescent cells [31,32,34,44]. Using our newly established method, senescence-escaped cells could be isolated shortly after senescence escape, allowing the early functional characterisation or further multi-omics investigation of senescence-escaped cells. Using this isolation method, MCF-7 and MDA-MB-231 cells after senescence escape were assessed to measure stem cell activity and proliferative and metastatic capacity. Different studies have described that senescence-escaped cells became more aggressive, exhibiting stem-like features and increased migration [21,29,30,31]. Our results indicated that the senescence-escaped cells regained the ability to proliferate, with restored proportion stem cell activity and migration capacity, resembling parental cell populations.

## 5. Conclusions

In conclusion, our research revealed that the senescence-escaping ability of therapy-induced senescent breast cancer cells depends on the cell type and senescence-inducing mechanism, and some senescent cells could escape senescence even after senolytic treatment. Our results also revealed that DPP4/CD26 could be a novel marker used to detect senescent breast cancer cells, which could be targeted using azithromycin treatment. The established FACS-based methods could contribute to a comprehensive investigation of senescence escape and the identification of potential therapeutic targets for decreasing senescence escape after breast cancer therapies.

## Data Availability

All data are available upon request.

## Supplementary Materials

The following supporting information can be downloaded at: https://www.mdpi.com/article/10.3390/cells13100841/s1, Figure S1: Evaluation of chromogenic (X-gal) SA-β-galactosidase staining using ImageJ software; Figure S2: Effect of bromodeoxyuridine (BrdU), gemcitabine (GEM) and palbociclib (PALBO) on the proliferation of MCF-7 and MDA-MB-231 cells; Figure S3: Representative figures of cell cycle analysis using PI staining; Figure S4: Representative images of BrdU-, GEM- and PALBO-induced senescent and senescence-escaped MCF-7 and MDA-MB-231 cells; Figure S5: DPP4/CD26 expression in BrdU-treated CAMA-1, MDA-MB-468 and T47D cells; Figure S6: Sitagliptin treatment in BrdU-, GEM- and PALBO-induced senescent MCF-7 cells; Figure S7: Representative images of the migration of control, senescent and senescence-escaped MCF-7 and MDA-MB-231 cells.
