# Supplementary material for: In Vitro Investigation of Therapy-Induced Senescence and Senescence Escape in Breast Cancer Cells Using Novel Flow Cytometry-Based Methods
Source: Cells. 2024 May 15;13(10):841. doi: 10.3390/cells13100841 (PMC11120107; doi:10.3390/cells13100841)
Supplement: Supplementary file 1 [file cells-13-00841-s001.zip › cells-2986406-supplementary.pdf]

Supplementary Figures

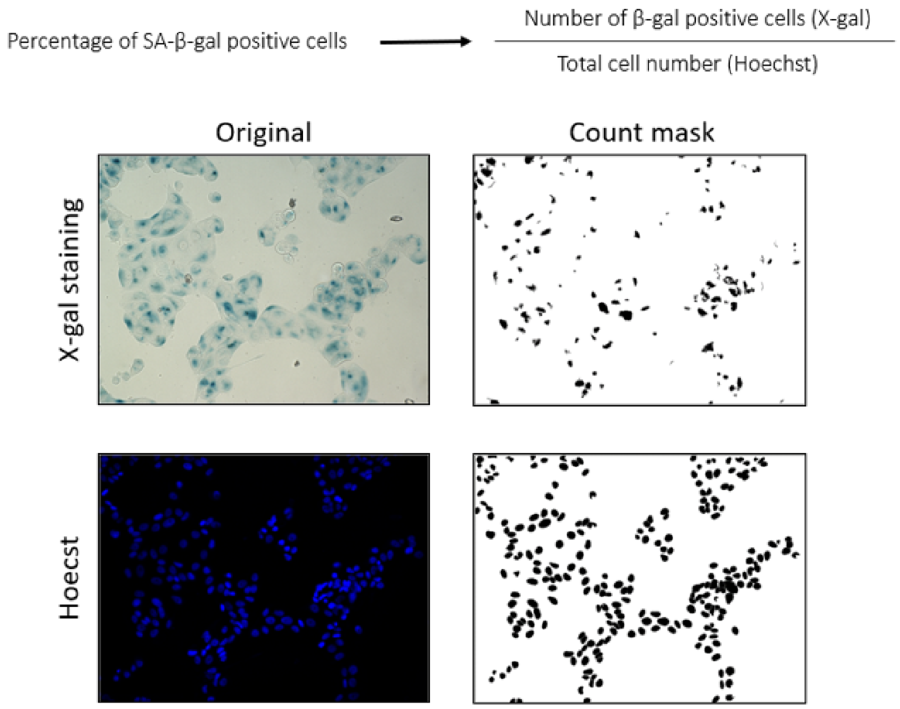

Figure S1. Evaluation of chromogenic (X-gal) SA-β-galactosidase staining by ImageJ software. Images on the left represent the original image of X-gal staining (brightfield) and Hoechst staining (DAPI channel) imaged by EVOS Auto Imaging System using magnification of 20X. Images on the right represent the count masks generated by setting up a colour/intensity threshold for positive staining by ImageJ software. The count masks were used to automatically count the positively stained cells by ImageJ software. SA-β-galactosidase staining was evaluated by using the formula presented above the images.

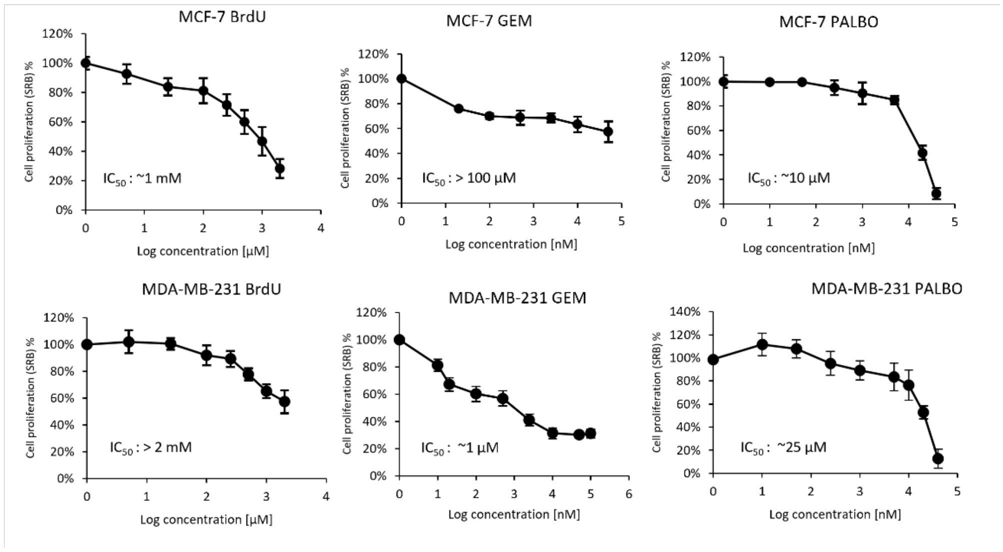

Figure S2. Effect of bromodeoxyuridine (BrdU), gemcitabine (GEM) and Palbociclib (PALBO) on the proliferation of MCF-7 and MDA-MB-231 cells. The proliferation of MCF-7 and MDA-MB-231 cells was measured by SRB assay after 72 hours treatment of BrdU, GEM and PALBO using different drug concentrations. Experiments were repeated three times with six technical replicates, values were normalised to vehicle-treated controls, error bars represent ± SEM.

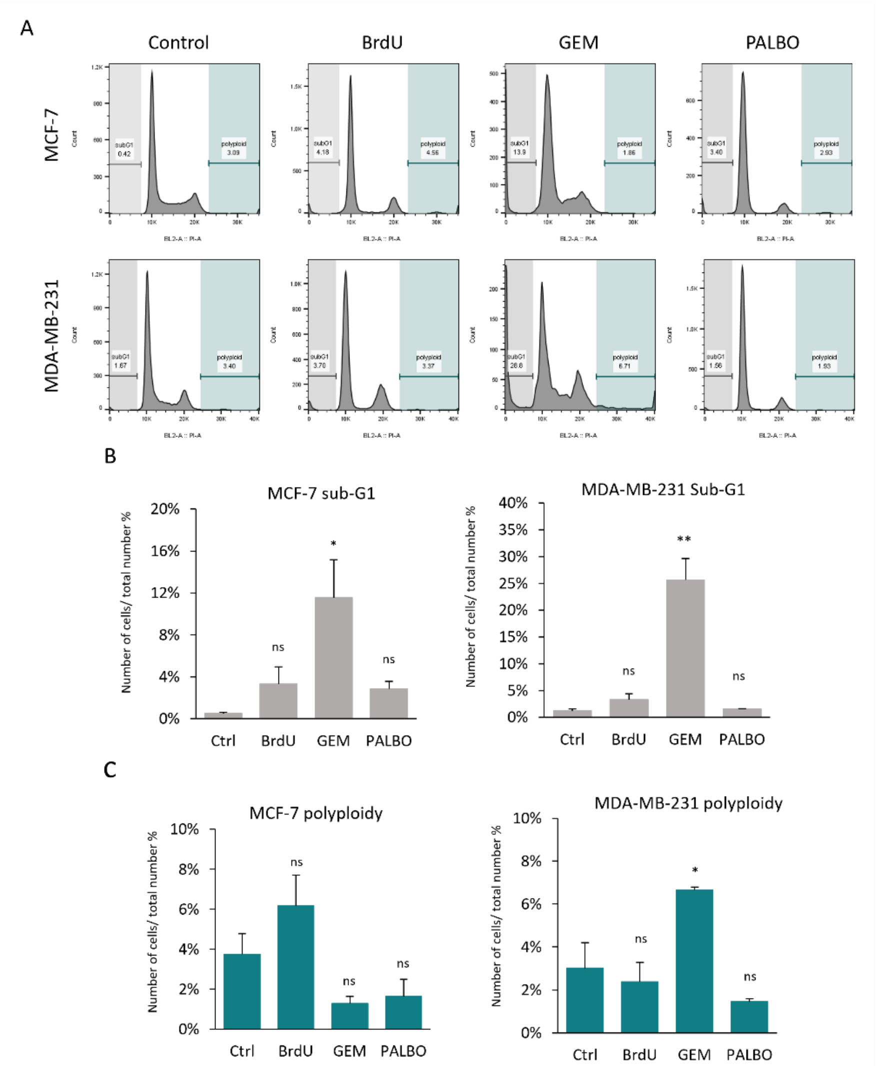

Figure S3. **(A)** Representative figures of cell cycle analysis using PI staining. The gates were adjusted manually to the sub-G1 and polyploid populations. The bar graphs represent the sub-G1 **(B)** and polyploid **(C)** populations in MCF-7 and MDA-MB-231 cells. Bar graphs represent the mean of three independent experiments ± SEM. Statistical significance (in relation to control): ns $p > 0.05$; * $p \leq 0.05$; ** $p \leq 0.01$.

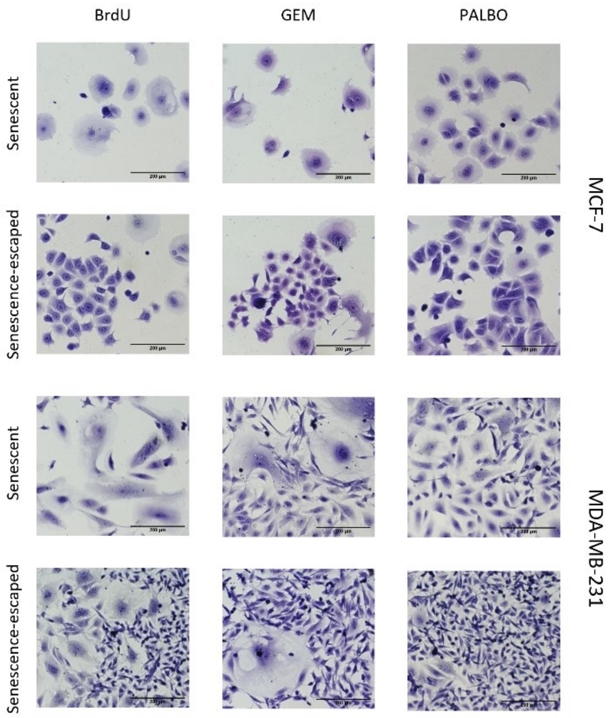

Figure S4. Representative images of BrdU-, GEM- and PALBO-induced senescent and senescence-escaped MCF-7 and MDA-MB-231 cells. The images indicate that PALBO-induced senescent cells have an increased ability to escape from senescence, compared to BrdU- and GEM-induced senescent cells. Cells were fixed and stained with crystal violet, imaged by EVOS with 10x magnification, scale bars indicate 200 µm.

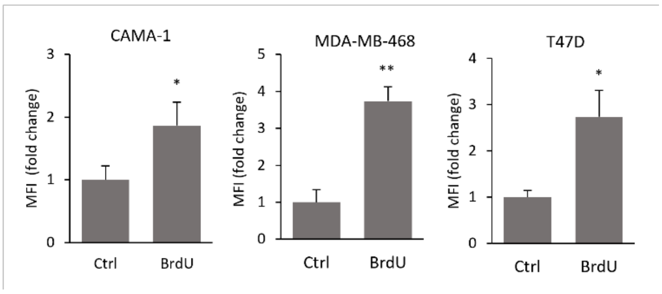

Figure S5. DPP4/CD26 expression in BrdU-treated CAMA-1, MDA-MB-468 and T47D cells. Cells were treated with 5 µM BrdU for 7 days. The expression of DPP4/CD26 in control and BrdU-treated cells was measured by flow cytometry. CD26 expression is represented as mean fluorescence intensity (MFI) and the bar graphs represent the mean of three independent experiments ± SEM.

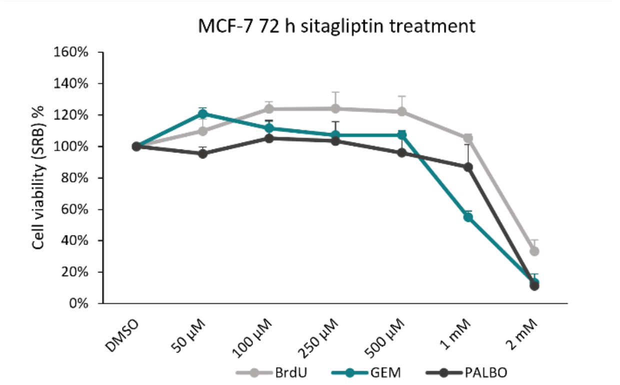

Figure S6. The cell viability of BrdU-, GEM- and PALBO-induced senescent MCF-7 cells was measured by SRB assay after 72 hours treatment of sitagliptin. Experiments were repeated three times with six technical replicates, error bars represent ± SEM.

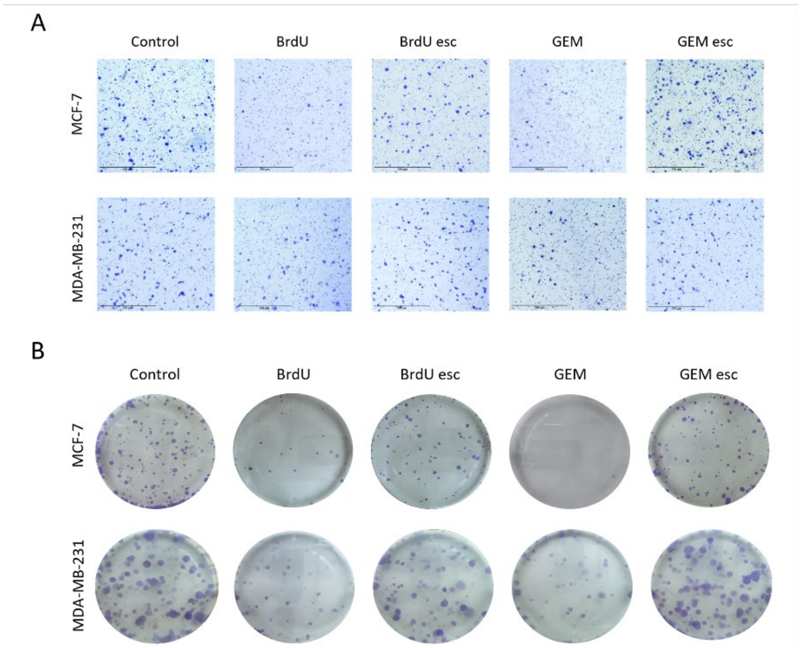

Figure S7. **(A)** Representative images of the migration of control, senescent and senescence-escaped MCF-7 and MDA-MB-231 cells. Cells were stained with crystal violet and imaged by EVOS using 10x magnification, scale bars indicate 750 µm. **(B)** Representative images of the colony formation of control, senescent and senescence-escaped MCF-7 and MDA-MB-231 cells. Cells were stained with crystal violet, and images represent the entire surface of a well of a 6 well plate.
